# Adjunctive treatment and BoNT-A for post-stroke spasticity: Are we really focusing on the patient-centered goals?

**DOI:** 10.3389/fneur.2023.1134691

**Published:** 2023-03-10

**Authors:** Alessio Baricich, Michele Bertoni, Andrea Santamato, Maurizio Osio, Giulio Gasperini, Alessandro Picelli, Franco Molteni, The Collaborative Working Group, Roberto Antonacci

**Affiliations:** ^1^Department of Health Sciences, Università del Piemonte Orientale, Physical Medicine and Rehabilitation, Maggiore della Carità University Hospital, Novara, Italy; ^2^Physical Medicine and Rehabilitation, ASST Settelaghi, Varese, Italy; ^3^Physical Medicine and Rehabilitation, Policlinico Hospital, Università di Foggia, Foggia, Italy; ^4^Department of Neurology, ASST Fatebenefratelli-Sacco, Milan, Italy; ^5^Villa Beretta Rehabilitation Center, Valduce Hospital, Costa Masnaga, Italy; ^6^Neuromotor and Cognitive Rehabilitation Study and Research Centre, Department of Neuroscience, Biomedicine, and Movement Sciences, University of Verona, Verona, Italy

**Keywords:** stroke, spasticity, rehabilitation, botulinum toxin type A, adjunctive treatment

## Introduction

Spasticity is a disorder of motor function that may follow upper motor neuron lesions after stroke, spinal cord injury, multiple sclerosis, or traumatic brain injury (1). Spasticity may cause decreased active movement, increased disability, and functional impairment in the affected patients.

If untreated, spasticity may cause secondary complications such as muscle and tendon contractures, joint deformity, a decrease in activities of daily living, pain, and other consequences, which may lead to a decrease in the quality of life (2–4). Botulinum toxin type A (BoNT-A) is a grade A effective and safe treatment for focal post-stroke spasticity (PSS) (5). Current evidence supports the effectiveness of BoNT-A in reducing muscle tone in both upper and lower limbs PSS (6–10). According to the Royal College of Physicians guidelines, spasticity management with BoNT-A aims to reduce symptoms, improve function, and prevent long-term consequences (11). BoNT-A also showed to reduce mispositioning of the limbs (12), improve posture and gait (8, 13, 14), reduce pain (15), reduce caregiver burden (16), and improve person-centered goals (17, 18).

However, several unmet needs are reported in the routine management of patients affected by post-stroke spasticity. Recent surveys addressed some of these issues, leaving other questions needing to be solved (19), such as optimal timing of treatment, the dose and muscle selection, and the ideal follow-up scheduling.

More recently, we suggested that BoNT-A intervention should be considered as soon as spasticity interferes with patients' clinical conditions (20). The authors also pointed out that monitoring of patients is required over time. Interestingly, this consensus also highlighted the relevance of patient-centered goals to answering patients' clinical needs, regardless of spasticity time onset and/or duration.

In addition, experts strongly agreed on the relevance of BoNT-A treatment goals, which may depend on several clinical challenges. It should also be pointed out that the goals and objectives of patients, caregivers, and medical teams must be carefully evaluated at initial evaluation, first treatment, and long-term management.

Indeed, post-stroke spasticity management must be part of a goal-oriented rehabilitation program focused on the patient's treatment goals. However, these goals are variable and may also change over time; for these reasons, our panel highlighted that both goal-setting and BoNT-A treatment schemes should be carefully reassessed at each follow-up visit.

The scientific literature reports the effectiveness of some adjunctive treatments to optimize the BoNT-A clinical effect; even though the previously published article did not focus on this topic, this aspect may be relevant for patients' goal attainment.

In the literature, two systematic reviews have looked into adjunctive therapies after BoNT-A in post-stroke spasticity. Mills et al. (21) concluded that there is high-level evidence to suggest that adjunct therapies may improve the outcomes following a botulinum toxin injection. By contrast, Kinnear et al. concluded that evidence related to the impact of adjunct therapy is available, but the heterogeneity of studies limits the opportunity to demonstrate the overall impact (22).

Current evidence does not clarify the role of each option in relation to patients' goals or long-term management of spasticity. However, considering the different focus of each adjunctive treatment on different aspects of upper motor neuron syndrome, clinicians should identify the best approach based on the individual needs of each patient affected by PSS and on their specific goals, considering all the aspects involved in this decision-making process.

Based on these considerations, this Delphi-based consensus survey aims to propose a rehabilitation management model that may include adjunctive treatments after BoNT-A, based on specific patient-centered goals, and that can support the clinicians in their clinical practice.

## Methods

A Delphi technique was used for this study in order to obtain consensus.

A three-round Delphi was proposed to generate pertinent items (round 1), explore preliminary consensus (round 2), and finally determine priorities based on levels of consensus achieved (round 3) (23).

The whole process was supervised and validated by a scientific committee of four experts in spasticity management (physical medicine and rehabilitation physicians), selected from the main Italian rehabilitation medicine societies (Italian Society of Physical and Rehabilitation Medicine, SIMFER, and Italian Society of Neurologic Rehabilitation, SIRN). The scientific committee identified the study participants (expert panel) and developed the material. A total of 20 physicians with experience in the field of spasticity management were invited to this survey. The participants/experts were required to be authors or co-authors of articles focused on “post-stroke spasticity and BoNT-A” published over the last 5 years.

A database was created using SPSS Statistics version 20 by IBM (Armonk, NY, USA). Percentages for each response were calculated. A proportion within a range method was used to define consensus. The definition of consensus was established before the data analyses. It was determined that consensus would be achieved if at least 75% of participants reached an agreement or disagreement. This level of agreement had been considered appropriate in previous Delphi studies (24, 25).

During the first round, the panel members received, *via* email, an open-ended question and were asked to provide answers accompanied by essential support of literature. The survey question was focused on the adjunctive treatment that should be considered for post-BoNT-A management in PSS. Based on the evaluations and feedback, a list of 12 possible treatments was identified (physiotherapy, stretching, strength exercises, antagonist muscles strengthening exercises, splint/orthosis management, caregiver counseling, robotic rehabilitation, electrical stimulation, taping, casting, occupational therapy, and extracorporeal shock waves therapy).

In the second round, all the experts were requested to express their level of agreement/disagreement (total agreement, partial agreement, and disagreement) on the use of these adjunctive treatments for both upper and lower limbs in different post-stroke phases as follows: (1) early post-acute phase (<3 months after stroke) for active goals; (2) early post-acute phase (<3 months after stroke) for passive goals; (3) post-acute phase (3–6 months after stroke) for active goals; (4) post-acute phase (3–6 months after stroke) for passive goals; (5) chronic phase (>6 months after stroke) for active goals; (6) chronic phase (>6 months after stroke) for passive goals.

Round 3 was an 8-h in-person session. Facilitated by an experienced independent moderator, the panel focused on the key aspects of the treatment paradigm for BoNT-A and adjunctive treatments for both upper and lower limbs in different post-stroke phases, taking into consideration some additional critical aspects, such as goal-setting definition, long-term management, and treatment adherence.

As stated in the Methods section, only the options which received the appropriate agreement rate were included in the following recommendations.

## Results

The expert panel identified different options based on different clinical phases of PSS and the specific patient-centered goals.

In the early post-acute phase, considering the active goals on the upper limb, the panel agreed on stretching, physiotherapy, strengthening exercises (including strengthening of antagonist muscles), and occupational therapy.

In the early post-acute phase, considering the active goals on the lower limb, the panel agreed on stretching, physiotherapy, and strengthening exercises (including strengthening of antagonist muscles).

In the early post-acute phase, considering the passive goals on the upper limb, the panel agreed on stretching, physiotherapy, and splint/orthosis management.

In the early post-acute phase, considering the passive goals on the lower limb, the panel agreed on stretching and physiotherapy.

In the post-acute phase, considering the active goals on the upper limb, the panel agreed on stretching, physiotherapy, strengthening exercises (including strengthening of antagonist muscles), occupational therapy, and robotic treatment.

In the post-acute phase, considering the active goals on the lower limb, the panel agreed on stretching, physiotherapy, strengthening exercises (including strengthening of antagonist muscles), and robotic treatment.

In the post-acute phase, considering the passive goals on the upper limb, the panel agreed on stretching, physiotherapy, and splint/orthosis management.

In the post-acute phase, considering the passive goals on the lower limb, the panel agreed on stretching, physiotherapy, splint/orthosis management, and caregiver's counseling.

In the chronic phase, considering the active goals on the upper limb, the panel agreed on stretching, occupational therapy, physiotherapy, strengthening exercises for antagonist muscles, and caregiver's counseling.

In the chronic phase, considering the active goals on the lower limb, the panel agreed on stretching, physiotherapy, caregiver's counseling, and strengthening exercises (including strengthening of antagonist muscles).

In the chronic phase, considering the passive goals on the upper limb, the panel agreed on stretching, caregiver's counseling, splint/orthosis management, and physiotherapy.

In the chronic phase, considering the passive goals on the lower limb, the panel agreed on stretching, splint/orthosis management, caregiver's counseling, and physiotherapy.

It should be noted that different approaches are proposed considering the goal-setting procedure for each person, together with the time since the stroke.

In particular, after the discussion, it was pointed out that harmful effects of spasticity classified according to the WHO ICF must be considered in order to tailor the rehabilitative interventions on impairment, activity, and participation—in fact, the personal characteristics of each patient might critically affect these aspects, based on the patient's motor and cognitive impairment.

In addition, systematic re-evaluation at each follow-up visit must consider a treatment adjustment that should include goal re-definition, target muscles, BoNT-A doses, and adjunctive treatments; clinical results of previous treatments must be considered in order to tailor the best treatment option at each stage.

## Discussion

The role of adjunctive treatment in the treatment of focal spasticity with BoNT-A has been extensively debated in the literature. Several articles have been published, and some reviews have attempted to clarify its effectiveness.

To the best of our knowledge, current evidence supports the use of adjunctive treatment in order to optimize the clinical effects of BoNT-A. However, on the contrary, more is needed to clarify the efficacy of each treatment by considering the importance of choosing the correct option for patients' needs.

In the previously cited document (20), an expert panel reached a broad consensus on the need to modify treatment schemes and goal identification with each treatment cycle in the initial evaluation and subsequent injections over the long term. However, as reported earlier, the role of rehabilitation and adjunctive therapies will not be addressed even if, in this perspective, the choice of adjunctive treatments must be taken into significant consideration.

This is a crucial point since rehabilitation treatment effectively improves the functional outcome of patients with stroke (26). In particular, some adjunctive treatments improved the passive characteristics of spastic muscles, whereas others also demonstrated a possible role in functional tasks such as gait speed (27).

Even with the limits of an excessive simplification linked to the schematization, distinguishing active functional objectives and passive objectives can facilitate the decision to include one or more of the additional treatments available in the rehabilitation project. It has been reported that most recovery after a stroke occurs 3–6 months after the event (28)—this aspect might be considered in order to identify the patient-centered goal for each phase better and, together with it, the adjunctive treatment that may best fit with this goal.

Finally, one of the most challenging aspects of long-term management is adherence to the treatment. As pointed out by Lee et al., at 5 years, <40% of patients with spasticity remained adherent—the reasons might include loss of interest due to the patient's symptoms not being sufficiently relieved by BoNT-A therapy and the patient's therapy goals not being achieved (29). On the contrary, greater adherence to therapy increased the odds of goal achievement for active indications, suggesting a possible interaction between the indication for injection and adherence to therapy (30).

In conclusion, we hypothesize that a correct choice of additional treatment based on the patient's needs is crucial in treatment adjustment to achieve the goal and optimize treatment adherence over time. Our results may aid clinicians in choosing the best option to provide optimal spasticity management.

Further research is needed to clarify the role of adjunctive treatments for the management of post-stroke spasticity with BoNT-A.

## The collaborative working group

Antonacci Roberto, Balestrieri Fabrizio, Battaglia Marco, Bissolotti Luciano, Borg Margherita, Campagnoli Marcello, Castagna Anna, Chisari Carmelo, Cinone Nicoletta, Corradini Claudio, Cosma Michela, Dalla Costa Davide, De Blasiis Paolo, Del Felice Alessandra, Morone Giovanni, Robecchi Majnardi Antonio, Sciarrini Francesco, Specchia Alessandro, and Trompetto Carlo.

## Author contributions

Conceptualization: AB, MB, and FM. Data curation, formal analysis, project administration, and writing—original draft: AB. Resources: AB, FM, AS, and AP. Supervision: MB, AS, MO, GG, and AP. Writing—review and editing: AP and AS. All authors have read and agreed to the published version of the manuscript.
